# Prognostic value of IRF-2 expression in colorectal cancer

**DOI:** 10.18632/oncotarget.17163

**Published:** 2017-04-17

**Authors:** Zubing Mei, Guanghui Wang, Zhonglin Liang, Ang Cui, Andong Xu, Yun Liu, Chenying Liu, Yili Yang, Long Cui

**Affiliations:** ^1^ Department of Colorectal Surgery, Xinhua Hospital, Shanghai Jiao Tong University School of Medicine, Shanghai, China; ^2^ Shanghai Colorectal Cancer Research Center, Shanghai, China; ^3^ Department of General Surgery, Second Affiliated Hospital to Yangzhou University School of Medicine, Yangzhou, Jiangsu Province, China

**Keywords:** colorectal cancer, interferon regulatory factor 2, prognosis, survival

## Abstract

Interferon regulatory factor 2 (IRF-2) is known to play a pivotal role in the development and progression of several malignancies. As a crucial member of interferon regulatory factor family, the association between the expression of IRF-2 and clinical prognostic significance has not been fully explored in colorectal cancer (CRC). The purpose of our study was to investigate the expression profile of IRF-2 in CRC and to examine its association with clinical features. The expression levels of IRF-2 in 18 paired CRC and non-cancerous colorectal tissues were measured by quantitative real-time PCR (qRT-PCR) and those in 4 paired samples by Western blotting. The results showed a significant increase in IRF-2 mRNA expression and protein expression in CRC tissues compared to those in paired normal tissues. Besides, high expression of IRF-2 was significantly associated with distant metastasis (*P* = 0.041) and preoperative serum CEA level (*P* = 0.045). Kaplan–Meier survival analysis showed that patients with high expression of IRF-2 had a significantly worse overall survival than those with low expression of IRF-2 (*P* = 0.006). Further multivariate analysis indicated that IRF-2 and TNM stage were independent prognostic factors for overall survival in patients with CRC. Our study primarily suggests IRF-2 as a potential prognostic biomarker in CRC.

## INTRODUCTION

As one of the most frequent malignancies, colorectal cancer (CRC) is the third most common cancer and the fourth most common cause of cancer death all over the world, with approximately 1.4 million people are diagnosed with CRC and 700,000 die of CRC annually [1]. Moreover, nearly 40 to 50 percent of newly diagnosed patients have developed or will progress to metastatic disease [2]. Although radical resection combined with adjuvant chemoradiotherapy has improved clinical outcomes for CRC, yet a high percentage of patients eventually suffer from local recurrence or distant metastasis. In the last few decades, numerous molecular markers have been exploited to detect CRC and predict outcomes, such as carcinoembryonic antigen (CEA) and carbohydrate antigen 19-9 (CA19-9) [3–6]. However, up till now, no specific or sensitive biomarkers have found to apply in clinical practice to predict and provide information for patient prognosis. In this case, there is an urgent need for identifying an effective biomarker to predict prognosis and guide postoperative treatment for patients with CRC.

Interferon regulatory factor 2 (IRF-2) belongs to one of the nine members of interferon regulator factor (IRFs) family, which has been reported to be associated with tumorigenesis and progression by activating gene transcription involving in oncogenesis such as histone H4 [7–10]. As its versatility in function, IRF-2 serves as a potential oncogene by inducing oncogenic transformation of NIH 3T3 cells [11]. In leukemic cells, IRF-2 was also identified as an inhibitor of activated N-RAS-induced growth suppression [12]. In several human malignancies, the differential expression of IRF-2 had been defined between tumors and adjacent non-cancerous tissues, such as breast [13], esophageal squamous cell [14], pancreatic [15] and hepatocellular cancer (HCC) [16], which was associated with clinical features and patient survival. Recent study by Chen et al. found the influence of miR-18a on the modulation of P53 expression by targeting IRF-2, which had a high predictive value for prognosis of gastric cancer patients [17].

Furthermore, IRF-2 has also been reported to play a bifunctional role in regulating tumorigenesis. Guichard et al found that in Hepatitis B Virus (HBV)-associated HCC, IRF-2 served as a tumor suppressor which regulated the p53 pathway. They hypothesized that lack of IRF-2 could impair p53 gene function [18].

Nevertheless, there is no evidence of large sample size of CRC patients to evaluate whether IRF-2 can act as a sensitive biomarker to predict the prognosis of CRC patients. Therefore, we investigated IRF-2 expression in 224 CRC patients and evaluated the possible relationship between IRF-2 expression and clinicopathological characteristics and prognosis in CRC by using immunohistochemistry.

## RESULTS

### Differential expression of IRF-2 in CRC tissues and paired adjacent normal tissues

**Figure 1:**
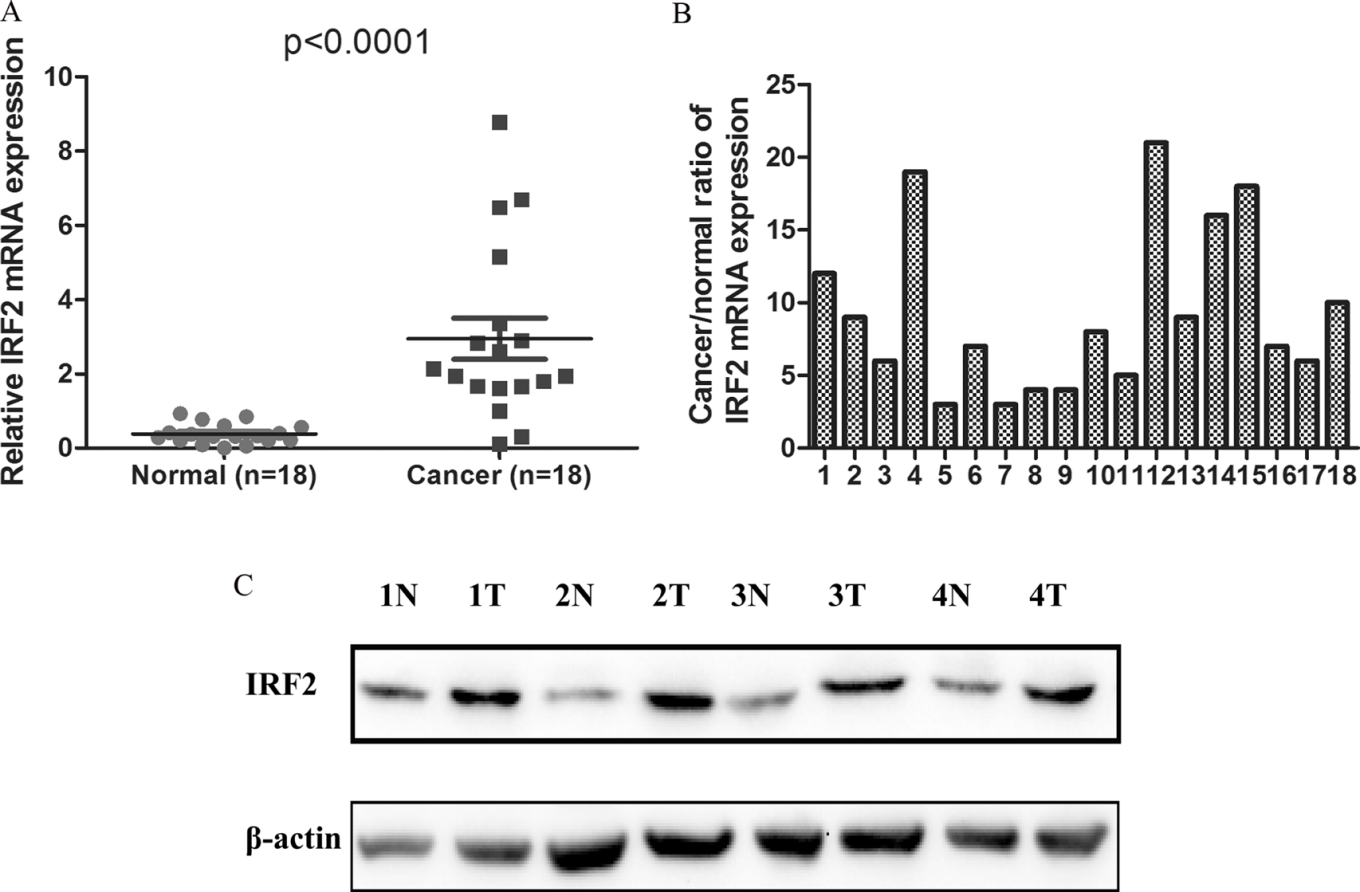
IRF-2 was up-regulated in colorectal cancer IRF-2 mRNA was markedly increased in tumor tissues than that in paired adjacent non-tumorous tissues (**A**, **B**). Western blotting analysis showed that IRF-2 protein expression was up-regulated in CRC tissues (T) when compared with paired non-tumorous tissues (*N*), β-actin was the loading control (**C**).

To better understand the mRNA expression pattern of IRF-2 in CRC tissues, qRT-PCR was performed in 18 pairs of tumor tissues and their adjacent non-tumorous tissues. We noted that IRF-2 mRNA expression was significantly up-regulated in CRC tissues (2.945 ± 0.553) when compared with paired adjacent normal tissues (0.384 ± 0.063) (*P* < 0.0001) (Figure 1A). The mean cancer/normal ratio of IRF-2 mRNA expression was 9.26 (range, 2.99 to 20.84) (Figure 1B). As shown in Figure 1C, increasing IRF-2 protein expression was noted in four CRC tissues compared with that in adjacent non-tumorous tissues, which was consistent with the result of qRT-PCR.

### Association between IRF-2 expression and clinicopathological characteristics

The association between clinicopathological characteristics and IRF-2 immunoreactivity is summarized in Table 1. High expression of IRF-2 was significantly associated with distant metastasis (*P* = 0.041) and preoperative serum CEA (*P* = 0.045) (Table 1). No significant differences were noted between IRF-2 expression and other clinicopathological variables such as age, gender, tumor site and size, TNM stage, tumor infiltration, lymph node metastasis, and differentiation, preoperative serum CA19-9 or histological type.

**Table 1:** Correlation between IRF-2 expression and clinicopathologic variables

Variables	IRF-2 expression			*P* value
	All cases	Low (%)	High (%)	
Age	224			0.298
≤ 70 ys	118	77 (65.3%)	41 (34.7%)	
> 70 ys	106	62 (58.5%)	44 (41.5%)	
Gender				0.251
Male	119	78 (65.5%)	41 (34.5%)	
Female	105	61 (58.1%)	44 (41.9%)	
Tumor site				0.130
Colon	112	64 (57.1%)	48 (42.9%)	
Rectum	112	75 (67.0%)	37 (33.0%)	
Tumor size (cm)				0.435
≤ 5 cm	97	64 (64.9%)	34 (35.1%)	
> 5 cm	127	76 (59.8%)	51 (40.2%)	
TNM stage				0.256
I–II	111	73 (65.8%)	38 (34.2%)	
III–IV	113	66 (58.4%)	47 (41.6%)	
Tumor infiltration				0.301
pT1-pT2	56	38 (67.9%)	18 (32.1%)	
pT3-pT4	168	101 (60.1%)	67 (39.9%)	
LN metastasis				0.556
pN0	181	114 (63.0%)	67 (37.0%)	
pN1-2	43	25 (58.1%)	18 (41.9%)	
Distant metastasis				0.041*
M0	186	121 (65.1%)	65 (34.9%)	
M1	38	18 (47.4%)	20 (52.6%)	
Serum CEA				0.045*
0–10 ng/ml	145	96 (66.2%)	49 (33.8%)	
> 10 ng/ml	71	37 (52.1%)	34 (47.9%)	
Serum CA19-9				0.528
0–20 u/ml	173	108 (62.4%)	65 (37.6%)	
> 20 u/ml	42	24 (57.1%)	18 (42.9%)	
Differentiation				0.605
Well	31	17 (54.8%)	14 (45.2%)	
Moderate	186	117 (62.9%)	69 (37.1%)	
Poor	7	5 (71.4%)	2 (28.6%)	
Histological type				0.083
Adenocarcinoma	200	128 (64.0%)	72 (36.0%)	
Mucinous/SRCC	24	11 (45.8%)	13 (54.2%)	

P*, χ^2^ test; SRCC, signet-ring cell carcinoma; p, pathologic stage; *P < 0.05.

### Prognostic value of IRF-2 in CRC

**Figure 2:**
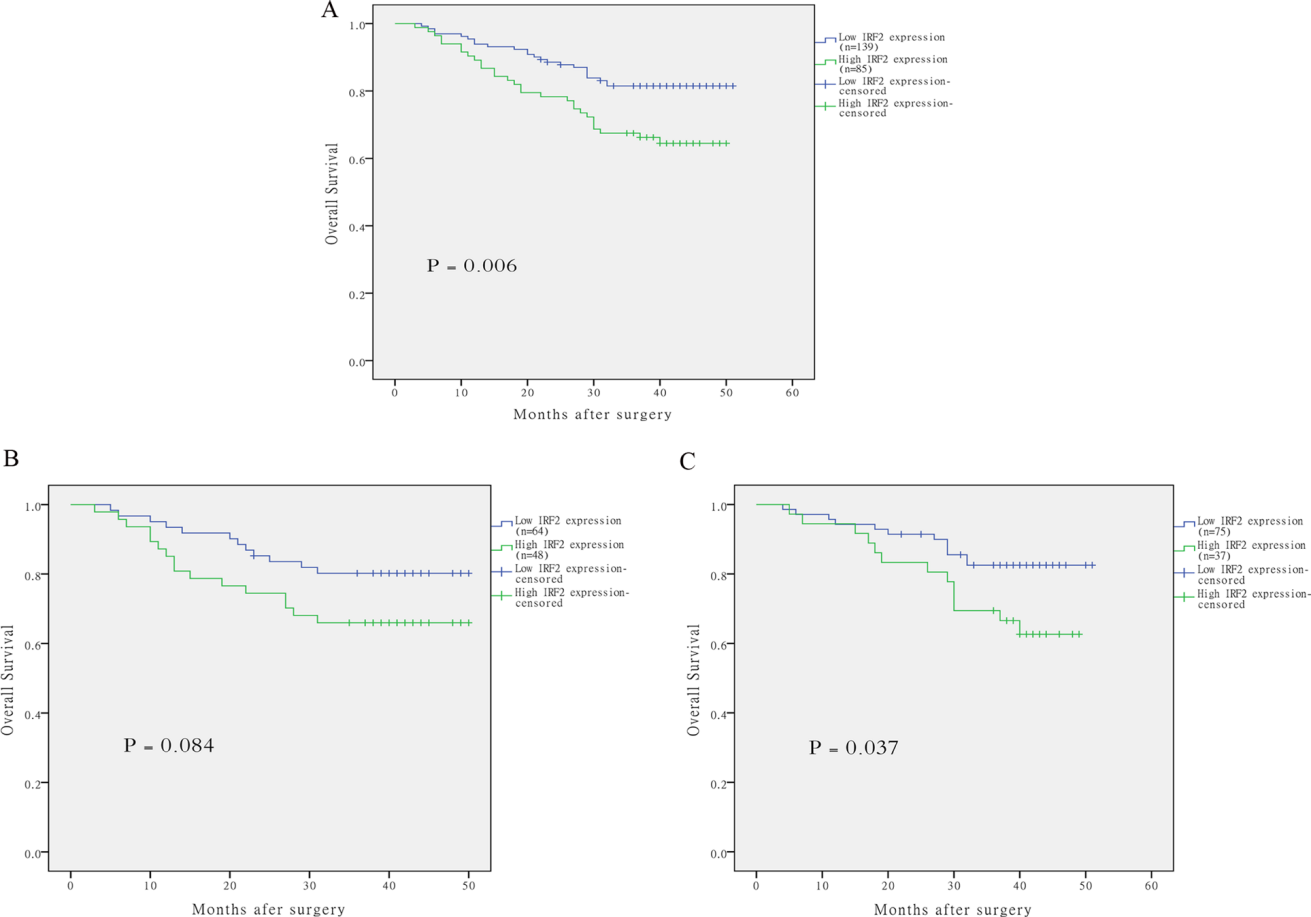
The correlation of IRF-2 expression and overall survival of patients with colorectal cancer Kaplan–Meier curves with univariate analysis revealed that patients with high expression of IRF-2 had a poorer overall survival than those with low expression of IRF-2 (**A**). Stratified analysis according to disease site revealed IRF-2 expression on overall survival was only pronounced in patients with rectal cancer (**C**), but not with colon cancer (**B**).

Results of Log-rank test indicated that patients with CRC of high IRF-2 expression tend to have worse overall survival with a mean overall survival of 39.2 months (95% CI 35.8 to 42.6), while those with low IRF-2 expression tend to have better overall survival with a mean overall survival time of 45.2 months (95% CI 43.0 to 47.2) (*P* = 0.006) (Figure 2A). Stratified analysis according to disease site revealed IRF-2 expression on overall survival was only pronounced in patients with rectal cancer (*P* = 0.037), but not with colon cancer (*P* = 0.084) (Figure 2B–2C). Univariate analysis showed that TNM stage, tumor infiltration, distant metastasis, lymph node metastasis, preoperative serum CEA and CA19-9 level together with IRF-2 expression were significantly associated with overall survival (Table 2). Multivariate analyses by using the Cox regression model revealed that IRF-2 expression as well as TNM stage was an independent prognostic factor for overall survival (hazard ratio 2.25, 95% CI 1.28 to 3.94, *P* = 0.005) (Table 3).

**Table 2:** Univariate analysis model for overall survival to assess IRF-2 expression in colorectal cancer and clinical features

Variables	Overall survival; Univariate HR (95% CI)	*P* value
Age (> 70 ys vs. ≤ 70 ys)	1.46 (0.85–2.51)	0.173
Tumor site (colon vs. rectum)	0.86 (0.50–1.48)	0.587
Tumor size (> 5 cm vs. ≤ 5 cm)	1.16 (0.27–2.00)	0.598
TNM stage (III–IV vs. I–II)	2.40 (1.36–4.23)	0.003*
Tumor infiltration (pT3-pT4 vs. pT1-pT2)	1.65 (0.83–3.28)	0.156
LN metastasis (pN1-2 vs. pN0)	1.91 (1.03–3.51)	0.039*
Distant metastasis (M1 vs. M0)	6.54 (3.78–11.33)	< 0.001*
Serum CEA (vs. 0–10 ng/ml)	2.30 (1.33–3.99)	0.003*
Serum CA19-9 (> 20 u/ml vs. 0–20 u/ml)	2.14 (1.15–3.96)	0.016*
Differentiation (poor vs. well/moderate)	1.08 (0.26–4.44)	0.914
Histological type (Mucinous/SRCC vs. adenocarcinoma)	0.65 (0.24–1.80)	0.408
IRF-2 expression (high vs low)	2.09 (1.22–3.59)	0.006*

HR, hazard ratio; 95% CI, 95 % confidence interval; LN, lymph node; p, pathologic stage; SRCC, signet-ring cell carcinoma; ys, years; *P < 0.05;Cancer staging was determined according to the 6th edition of AJCC cancer staging manual.

**Table 3:** Final multivariate analysis model for overall survival to assess IRF-2 expression in colorectal cancer and clinical features

Variables in the final model	Overall survival; Multivariate HR (95% CI)	*P* value
TNM stage (III–IV vs. I–II)	2.70 (1.49–4.89)	0.001*
IRF-2 expression (high vs low)	2.25 (1.28–3.94)	0.005*

HR, hazard ratio; 95% CI, 95 % confidence interval; *P < 0.05; Cancer staging was determined according to the 6th edition of AJCC cancer staging manual.

## DISCUSSION

As regulators of the type I interferon (INF) system, interferon regulatory factors (IRFs) have been recognized as crucial transcription factors that modulate host defense such as innate and adaptive immune response [19–22]. The IRF family can be classified into nine distinct groups, designated IRF-1 to IRF-9. It has been identified that these versatile factors participate in the regulation of cell growth, differentiation and apoptosis, which is associated with oncogenesis and tumor progression [19, 20, 22–24]. IRF-2 was initially considered as a negative regulatory protein which attenuated the genetic transcription of IFN-α and IFN-β gene induced by interferon-sensitive response elements [25, 11]. Further studies have indicated that overexpression of IRF-2 can lead to carcinogenic transformation in NIH 3T3 cells in nude mice [11].

In the last two decades, the multifunctional IRF-2 in oncogenesis and altered expression in several human malignancies have been reported and this differential expression may influence clinical outcomes of cancer patients. Yi et al found that high expression of IRF-2 was associated with increased recurrence and shorter overall survival in patients with HCC [16]. Doherty et al found that high-grade breast ductal carcinoma *in situ* and invasive ductal cancers were much more likely to express the oncogenic IRF-2 protein than the normal tissues [13]. They identified that the development of IRF-2 expression was correlated with oncogenic activation in breast cancer. Those studies reported that IRF-2 promoted oncogenesis by antagonizing IRF-1 in those tumors. Passioura et al found that overexpression of IRF-2 could inhibit mutant N-ras-induced growth suppression of myeloid cells which affected the development of murine models of human acute myeloid leukemia [12]. Moreover, IRF-2 has been found to modulate the growth of pancreatic cancer cells by regulating proliferation and apoptosis effectors, such as cyclin D1 and BAX [15]. However, high expression of IRF-2 in ovarian cancer patients was reported to be associated with improved disease-free and overall survival [26]. These conflicting findings led us to investigate IRF-2 expression in human CRC tissues and its impact on patient survival.

In our study, real time RT-PCR analysis demonstrated an approximate 9-fold of mean increase in IRF-2 mRNA level in CRC compared with that in adjacent normal tissues. Western blot analysis also confirmed the increased IRF-2 protein expression in CRC, which was consistent with the results of immunohistochemistry. These results conformed closely to those reported in pancreatic cancer [15], esophageal cancer [14] and breast cancer [13], further providing evidence that IRF-2 overexpression might be associated with tumorigenesis of CRC. Besides, high expression of IRF-2 was significantly correlated with some important clinicopathological variables such as distant metastasis, indicating its potential closely relationship with oncogenic feature of IRF-2.

Although currently the underlying mechanisms are still unclear, the role of IRF-2 could be hypothesized through analysis of IFN-mediated pathways in which IRF-2 was involved. The pro-oncogenic function of IRF-2 can be mediated by other IRFs through transcriptional interference, which can connect with some shared sequences involving in the initiation and progression of tumors. Chae et al found that IRF-2 enhanced NF-κB activity through the nuclear recruitment of NF-κB, thus contributing to the oncogenic potential of IRF-2 [27]. The fact that IRF-2 expression was often elevated in cancer cells, and elevated IRF-2 levels would enhance NF-κB activity when delivered an activation, such as TNF-α. On the other hand, IRF-2 expression could influence the survival of malignant cells exposed to such microenvironment. They proposed for further study to elucidate the role of IRF-2–NF-κB interaction on tumorigenesis, progression and drug resistance. Chen et al. found that forced expression of miR-18a could downregulate IRF-2 expression and inhibit P53 expression, indicating that IRF-2 could serve as a tumor suppressor by regulating P53 signaling in gastric cancer [17].

Univariate analysis for our study demonstrated that high expression of IRF-2 was one of the most significant prognostic variables for CRC and correlated with more than a two-fold increase in risk of all-cause mortality. Multivariate analysis further confirmed the role of IRF-2 regarding overall survival for CRC patients, which was independent of TNM stage and other factors. Current results underscored the prognostic value of IRF-2 in CRC patients.

This study has specific strengths. To the best of our knowledge, our study is the first one investigating the association between IRF-2 expression and its clinicopathological features as well as its prognostic significance in patients with CRC. The relative homogenous patient characteristics largely reduced the confounding effects on clinical outcomes. Our preliminary results revealed that IRF-2 was up-regulated in CRC tissues, which was significantly correlated with distant metastasis and preoperative serum CEA level, as well as a poor overall survival in patients with CRC based on our study cohort. Moreover, the predictive role of IRF-2 on patient prognosis was independent of TNM stage and other clinical variables.

Due to lack of molecular prognostic markers, new molecular markers for CRC are urgently needed in clinical practice and decision making, particularly for the purpose of implementing targeted therapy and improving patient survival. Up till now, the mechanism of IRF-2 in CRC is still poorly understood. Therefore, further studies are required to verify the molecular mechanisms regulating IRF-2 expression and its direct downstream transcriptional targets.

Several other potential limitations of this study should be addressed. Firstly, limited number of the patients in our study were enrolled, which reduced the statistical power in stratified analysis. Secondly, the absence of measurement of other IRF family members, such as IRF-1, might have also influenced the findings of this study because they could have interactive effect on tumor progression. Thirdly, the retrospective collection of the patient information is another major limitation of this study because recall bias may occur, although we try our best to minimum this bias. In future study, we should prospectively enroll larger number of CRC patients and collect more detailed information to further confirm the relationship between IRF-2 expression and CRC prognosis. Finally, as the data collection was retrospective in study design, some important survival information, such as the cause of death, the recurrence or progression date of many patients were missing, thus many other outcome measures, such as cancer-specific survival, recurrence-free survival could not be obtained for analyses. However, future study should be prospectively designed to add these information during follow-up.

In summary, our findings suggest that IRF-2 may be a valuable molecular biomarker for predicting the prognosis in CRC patients and serve as a potential therapeutic target for CRC. Furthermore, our findings also suggest a potential therapeutic target by regulating IRF-2 expression to reduce the malignant progression of CRC and to help benefit survival in CRC patients.

## MATERIALS AND METHODS

### Patients and tissue samples

A consecutive of 224 paraffin-embedded human CRC tissues and corresponding adjacent non-cancerous tissues were obtained from patients who had undergone radical tumor resection at the Department of Colorectal Surgery, Xinhua Hospital, Shanghai Jiao Tong University School of Medicine between 2008 and 2010. A total of 118 men and 106 women were included in this study, aged from 26 to 92 years (mean, 63.7 ± 14.2 years). There were 112 (50%) colon cancers and 112 (50%) rectal cancers, 111 of which were stage I/II disease and 113 were stage III/IV disease. All the included patients did not receive chemoradiotherapy prior to surgery. Table 1 presented the basic clinicopathological features of all included patients.

During a mean follow-up time of 36.4 months (range 3 to 51 months), 53 of 224 patients died. Outpatient visit combined with telephone interview was performed to evaluate the follow-up status and updated once every three months. This study was approved by the Ethics Committee of clinical review board.

Overall survival was defined as the time from radical resection to death irrespective of any occurrence of death. The clinical data were retrieved from the hospital-based database, including patient age, gender, tumor site and size, TNM stage, preoperative serum CEA and CA19-9 level, tumor differentiation, histological type and follow-up status.

### RNA extraction and quantitative RT-PCR (qRT-PCR) analyses

Total RNA was extracted from frozen tissues using TRIzol reagent (Invitrogen) according to the manufacturer's protocol. Concentration of RNAs was estimated by measuring the absorbance at 260 nm in spectrophotometer. One microgram of each RNA sample was reverse-transcribed to cDNA using PrimeScript reverse transcriptase (TaKaRa) and qRT-PCR was performed on the ABI 7500 Real-Time PCR system using SYBR Premix Ex Taq (TaKaRa) to detect IRF-2 mRNA expression based on the manufacturer's instructions. The primers to IRF-2 were designed as follows: forward, 5′-TGGATGCATGCGGCTAGA-3′; reverse, 5′-CATCTGAAATTCGCCTTCC-3′. β-actin served as an internal control, its primers were as follows: forward, 5′-GATCATTGCTCCTCCTGAGC-3′; reverse, 5′-ACTCCTGCTTGCTGATCCAC-3′.

### Western blotting

Western blotting procedure was carried out according to the manufacturer's instructions. Briefly, frozen tissue samples were crushed and ground into fine powder in liquid nitrogen and lysed with 10% SDS-PAGE and transferred onto polyvinylidene difluoride (PVDF) membranes. After blocked with 5% fat-free dry milk for 1 hour, the membranes were incubated with anti-human IRF-2 antibody (1:1000, Santa Cruz Biotechnology, Santa Cruz, CA) or β-actin antibody (1:1000, Epitomics, Burlingame, USA) overnight at 4°C. The next day after washing with TBST buffer, horseradish peroxidase (HRP)-conjugated goat anti-rabbit IgG (1:5000, Santa Cruz, CA) for 1 hour, and IRF-2 expression was detected using ECL prime Western blotting detection reagent (Amersham). β-actin acted as a loading control.

### Immunohistochemistry (IHC) and scoring of IRF-2 immunostaining

IHC was performed using the standard protocol. In brief, 4 μm-thick formalin-fixed, paraffin-embedded sections were deparaffinized with three changes of xylene, and rehydrated with decreasing graded ethanol. Antigen retrieval was performed by boiling sections for 30 minutes at 95°C in pH 6.0 (0.01 m) sodium citrate buffer. Endogenous peroxidase activity was blocked by immersing the sections in 3% hydrogen peroxide for 10 minutes. And then the slides were incubated with antibody against IRF-2 (no. sc-13042, 1: 100 dilution; Santa Cruz Biotechnology, Santa Cruz, CA) overnight at 4°C. After rinsing with PBST, goat anti-rabbit biotinylated antibody (GK500710; Gene Company Ltd., Shanghai, China) was used and incubated for 30 minutes at room temperature. Slides were developed with 3,3′-diaminobenzidine (Sigma, St. Louis, MO) (DAB) and counterstained with hematoxylin, dehydrated, cleared, and coverslipped.

**Figure 3:**
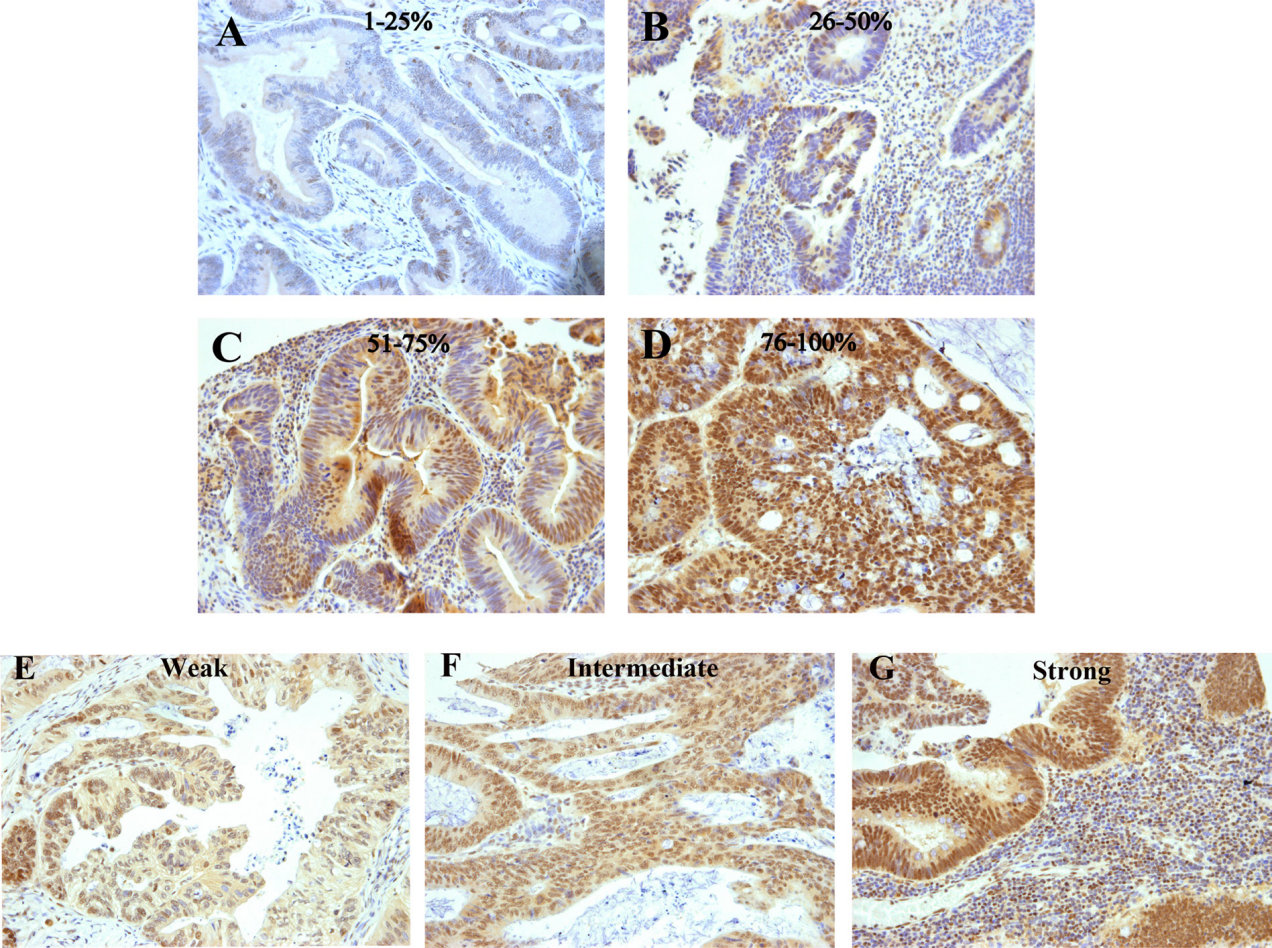
Examples of colorectal cancers immunostained for IRF-2 Various percentages of IRF-2-positive cells are exemplified in the upper rows (**A**–**D**) and various IRF-2 staining intensities are exemplified in the lower row (**E**–**G**).

IRF-2 expression was evaluated and scored by two independent investigators who were blinded to clinicopathological and survival data. We applied a revised semi-quantitative scoring system based on previous studies [14, 15], as follow: staining intensity was scaled as 0 (absent staining), 1 (weak staining), 2 (intermediate staining) and 3 (strong staining) (Figure 3E–3G). The percentage of immunoreactive cells was designated as 1 (< 25%), 2 (25–50%), 3 (51–75%) and 4 (>75%) (Figure 3A–3D). For each section, the final score for IRF-2 expression was the product of percentage of immunoreactive cells and the intensity score ranging from 0 to 12. Thus, IRF-2 expression was dichotomised into high or low with a score less than 6 defined as low expression and a score equal or more than 6 as high expression. Any discrepancies were discussed by the original two investigators and a senior pathologist until a consensus was reached.

### Statistical analyses

All statistical analysis was conducted using IBM SPSS Statistics version 19 (IBM Corp., Armonk, NY). The results of qRT-PCR were expressed as mean ± SD. The association between IRF-2 expression scaled by immunohistochemistry and clinicopathological features was analysed using χ^2^ test or a two-sided Fisher's exact test. The Kaplan–Meier method was applied to estimate patient overall survival. The log-rank test was performed to evaluate the survival differences between subgroups. Univariate and multivariate logistic regressions were used to assess the association between IRF-2 expression and overall survival. The independent prognostic factors for overall survival were examined using the Cox proportional hazards models for the multivariate analysis, which were applied to estimate hazard ratios for mortality. The initial time point for the survival modeling was the date of radical resection of tumor. Patients were censored if they survived by the end of the follow-up period. The proportional hazards assumption was checked both graphically by inspecting the log (−log) plots of the survival function, as well as using the Schoenfeld residuals and associated test statistics [28]. To model the effect of IRF-2 expression in a way that allows for the fact that exposure is varying over time, it is necessary to assess IRF-2 expression for each surviving patient at every point in time at which an event (e.g., death) occurs. Therefore, we tested the proportional hazards assumptions by employing the methodology of time-dependent covariates and found to be appropriate. A multivariable analysis with a stepwise Cox regression model of some selected baseline characteristics was conducted to test the effect of IRF-2 expression after adjustment for the statistically significant prognostic factors obtained by univariate analysis. A two-tailed *P* value of less than 0.05 indicated statistically significant.
